# Detection of Lipid and Amphiphilic Biomarkers for Disease Diagnostics

**DOI:** 10.3390/bios7030025

**Published:** 2017-07-04

**Authors:** Jessica Z. Kubicek-Sutherland, Dung M. Vu, Heather M. Mendez, Shailja Jakhar, Harshini Mukundan

**Affiliations:** 1 Physical Chemistry and Applied Spectroscopy, Chemistry Division, Los Alamos National Laboratory, Los Alamos, NM 87545, USA; jzk@lanl.gov (J.Z.K.-S.); dvu@lanl.gov (D.M.V.); sjakhar@lanl.gov (S.J.); 2 Department of Chemical and Biological Engineering, University of New Mexico, Albuquerque, NM 87131, USA; hmendez@unm.edu; 3 The New Mexico Consortium, Los Alamos, NM 87544, USA

**Keywords:** biomarkers, biosensors, amphiphile, lipid, diagnostics

## Abstract

Rapid diagnosis is crucial to effectively treating any disease. Biological markers, or biomarkers, have been widely used to diagnose a variety of infectious and non-infectious diseases. The detection of biomarkers in patient samples can also provide valuable information regarding progression and prognosis. Interestingly, many such biomarkers are composed of lipids, and are amphiphilic in biochemistry, which leads them to be often sequestered by host carriers. Such sequestration enhances the difficulty of developing sensitive and accurate sensors for these targets. Many of the physiologically relevant molecules involved in pathogenesis and disease are indeed amphiphilic. This chemical property is likely essential for their biological function, but also makes them challenging to detect and quantify in vitro. In order to understand pathogenesis and disease progression while developing effective diagnostics, it is important to account for the biochemistry of lipid and amphiphilic biomarkers when creating novel techniques for the quantitative measurement of these targets. Here, we review techniques and methods used to detect lipid and amphiphilic biomarkers associated with disease, as well as their feasibility for use as diagnostic targets, highlighting the significance of their biochemical properties in the design and execution of laboratory and diagnostic strategies. The biochemistry of biological molecules is clearly relevant to their physiological function, and calling out the need for consideration of this feature in their study, and use as vaccine, diagnostic and therapeutic targets is the overarching motivation for this review.

## 1. Introduction

The utility of a diagnostic is measured by its ability to provide rapid and reliable information to guide treatment. The past century has seen a rise in molecular diagnostic strategies for measuring disease-specific signatures in patient samples (e.g., blood, urine, tissue and others) using a variety of biosensor technologies. A biosensor utilizes a biological component or biocatalyst to detect the presence of an analyte and a transducer to create a quantifiable signal from this interaction. For a biosensor to be used feasibly in a clinical setting, it must be highly specific for the target analyte, accurate in patient samples, rapid and reliable, and resistant to non-specific interactions in clinical samples. In addition, for some applications, especially in resource-poor conditions, it is also desirable for the sensor to be cost-effective and easy-to-use [1]. In order to avoid false positive (signal in the absence of analyte) and false negative (analyte present without signal) signals, the biocatalyst and transducer must be carefully considered for each target analyte.

Identifying target analytes and their presentation in a host during disease is of crucial importance for the success of such molecular diagnostic technologies. Biomarkers are indicators of biological processes and disease-specific biomarkers are of great clinical value for diagnostics. Understanding the interactions between the biomarker and its host is key to developing assays for the detection of such analytes in clinical samples. Because of the ease of purification and detection, proteins and nucleic acids have been extensively used as diagnostic targets. Traditionally, nucleic acids and proteins have been targeted as biomarkers for the diagnosis and detection of disease, primarily because of the availability of a variety of sensitive and tailored methods for the detection of these biochemical signatures (reference). However, another category of biomolecules that play important roles in a variety of cellular processes, and can therefore serve as indicators of disease and analytes for biosensors is lipids and amphiphiles [2]. Besides membrane formation, lipids also play a crucial role in cell signaling (steroids) and energy storage. The brain is composed of 40%–81% lipid, with the myelin sheath being 78%–81% lipidic in biochemistry [3]. Many of the bacterial virulence factors recognized by the human innate immune response, such as lipopolysaccharide (LPS) and lipoteichoic acid (LTA), are amphiphilic lipoglycans [4,5,6,7,8,9,10]. The structure of lipids is far more diverse than that of proteins, nucleic acids or carbohydrates, allowing for highly specific biomarkers for a variety of diseases. However, all lipids contain at least one hydrocarbon chain that is insoluble in water making them difficult to detect in aqueous solutions such as blood, which has largely limited their application as diagnostic targets. Indeed, currently, rapid detection of lipidated targets is largely achieved via methods that were originally designed for proteins. Here, we review advancements in methods for detection of lipid and amphiphilic biomarkers associated with both infectious and non-infectious diseases describing the advantages and limitations of each approach in clinical diagnostics (Table 1).

## 2. Methods for Detecting Lipid and Amphiphilic Biomarkers

### 2.1. Storage and Processing of Patient Samples

Sample collection and preparation is a critical consideration for the detection any biomarker [11], but lipidic targets warrant further attention [12]. Common lab methods such as freeze/thaw and chemical extractions can have a profound effect on the viability of amphiphilic biomarkers [13,14]. Further, depending on the sample, lipid and amphiphilic biomarkers are often sequestered by host factors, further reducing their availability for direct detection [15]. Therefore, any biosensor designed to detect amphiphilic biomarkers must account for their unique biochemical properties.

### 2.2. Mass Spectrometry

Lipidomics requires a thorough characterization of the structure and the function of lipids within a living system. The study of amphiphilic biomarkers, as described here, is a composite of lipidomics, but involves the consideration of the hydrophilic components of the biomarker in addition to the hydrophobic lipids. Since its inception in the early 20th century, mass spectrometry (MS) has played a significant role in characterization of lipids [16,17,18], and this has been extensively reviewed. Here, we will focus on the adaptive application of MS to amphiphilic biomarkers.

MS ionizes lipids and sorts ions based on their mass-to-charge ratio. It has been widely used to characterize lipids [19,20,21,22,23], especially with the development of soft ionization techniques such as electrospray ionization (ESI) and matrix-assisted laser desorption ionization (MALDI). Lipid extraction is usually the first step for lipid analysis, and separates the lipidic components (organic phase) from other components such as proteins and nucleic acids (aqueous phase). Most widely used extraction methods have been adapted from Folch [24] or Bligh and Dyer [25], in which a mixture of methanol, chloroform and water are applied for phase separation. However, shotgun lipidomic methods have also been developed which omit the chromatographic separation and sample processing described above, and analyzes all lipid classes together, instead using ionization additives to provide discriminative identification [16]. This method might be more suitable especially for biomarker-discovery, wherein there is no prior art regarding the biomarkers being identified/measured.

Chromatographic methods such as gas chromatography, thin-layer chromatography, high-performance liquid chromatography (HPLC), or ultra-performance liquid chromatography (UPLC) [21,26] are used for separation of lipid mixtures. HPLC and UPLC are have broader applications in lipid analysis [20,26] and can be performed in normal phase (lipid class separation based on their different polarities and dipole moments) or reverse phase (separates lipid according to their hydrophobicity and is based on fatty-acyl compositions). Coupling of separation with LC methods allows lipids to be resolved sequentially with greater ionization yields, decreased ion suppression from major lipid classes, and increased sensitivity [20,27]. However, this LC step can be bypassed, with direct infusion (shotgun lipidomics) of lipid samples, which simultaneously analyzes the lipid species in the crude lipid extract [28,29]. This method does not require a priori decisions on which lipid species to measure, is relatively simple, high-throughput, and fast with short data acquisition times. However, a major limitation of this method is that highly abundant lipids can compete for ionization with the minor species, and mask detection of the latter.

MALDI-MS is based on the utilization of an ultraviolet–absorbing matrix that initially absorbs the energy of the laser and mediates the generation of ions [30,31]. ESI-MS uses a high voltage electrospray to aerosolize the lipid sample and generate ions to charge the lipid molecule [19,32]. MALDI or ESI ionization can be combined with several types of mass analyzers such as triple quadrupole, time-of-flight (TOF), ion trap, and orbitrap to further characterize different lipid classes and species [32,33]. Both quadrupole and TOF mass analyzers are commonly used and their configuration together (e.g., triple quadrupule (QqQ), quadrupole time-of-flight (QTOF)) as tandem mass spectrometric instruments to break down the precursor ions into fragmented product ions, can provide further chemical and structural resolution on the lipid species [34,35]. As an example, ESI-MS has been successfully applied to characterize cardiolipin [35]. Cardiolipin, a biomarker implicated in heart disease and cancer, is a phospholipid present in mitochrondria and is a component of bacterial membrane [36,37]. The diversity of cardiolipin molecular species is found in both the identity and position of its four fatty acyl moieties. Minkler and Hoppel showed that reverse-phase ion pair HPLC coupled with a triple quadrupole MS/MS linear ion trap mass spectrometer to generate MS/MS was highly effective in characterizing cardiolipin from different species (e.g., rat liver, mouse heart, bovine heart, dog heart) [35], with the spectra facilitating deduction of the structure of cardiolipin: the diacylglycerol phosphate region, the monoacylglycerol region, and the fatty acid region [35].

Mass spectrometry has been used by clinical laboratories to rapidly diagnose both infectious and non-infectious diseases, detect drug toxicity, monitor treatment as well as to discover new biomarkers [38]. However, clinical mass spectrometry systems such as VITEK^®^ MS by BioMerieux are expensive to set up and maintain and require trained personnel to perform and analyze the tests limiting its utility as a point-of-care diagnostic tool.

### 2.3. Nuclear Magnetic Resonance (NMR)

NMR spectroscopy is one of the most powerful analytical techniques for lipid analysis of biological matrices (e.g., cells, tissue, and biological fluids), owing to the natural abundance of hydrogen in such samples [39,40,41,42,43]. This method is nondestructive, nonselective, and provides detailed molecular information, which can be of extensive value in analyzing amphiphilic biomarkers. Specifically, high-field proton nuclear magnetic resonance (^1^H-NMR) spectroscopy is used for molecular profiling [44,45], allowing for identification of biomarkers associated with diseases such as tuberculosis [46], cancer [47,48], heart disease [49]; and others, identifying underlying causes of disease [50], and identifying diagnostic biomarkers and new therapeutic strategies [51]. High-resolution, one-dimensional (1D) ^1^H NMR is reproducible and simple [39,49]. It has been applied for metabolomics since the 1980s [39,42], specifically, for plasma [48], serum [52], urine [43], and feces [53]. Tissues are normally examined intact [54] and spectra can be obtained rapidly (<5 min) [39] with detection limits around 1 to 10 µM at > 500 MHz [42,50] MS and NMR are often used in conjunction with one another [55,56]. Although NMR has decreased sensitivity in comparison to MS [50,57], it has other advantages such as being a non-destructive technique with minimal sample preparation, and providing quantitation of molecular structures [18,57]. Coupling the two method yields significantly greater sensitivity (attomole to femtomole) [58].

Lipidomic NMR is associated with many challenges. A substantial number of biomolecules in complex matrices have similar resonances, due to similar proton chemical shift ranges [42,59] resulting in considerable peak overlap [46,60] and a low signal-to-noise ratio [61]. Minimizing peak overlap and increasing sensitivity can be resolved by using high resolution spectrometers (800 to 950 MHz) [62]. Resonance line broadening [63] attributed to the formation of micelles in an aqueous environment or constrained molecular movement as a result of lipid aggregation into bilayers [42] is another challenge that is overcome by chemical or physical treatment of the sample [42].

The use of two-dimensional (2D) NMR [64,65] can also overcome the above challenges of 1D ^1^H NMR. 2D techniques such as the heteronuclear single quantum coherence (HSQC) method are better suited for lipid profiling because of its ability to detect resonances for both ^1^H nuclei and ^13^C nuclei [61], thus allowing for correlation between ^1^H and ^13^C chemical shifts and providing elucidation of C-H bonds within a structure [50]. Although detection of ^13^C is possible with HSQC, the natural abundance of ^13^C [66] is relatively low, requiring prior enrichment of cells with ^13^C [46]. 2D NMR is more time consuming (>1 h/spectrum) in comparison to 1D ^1^H NMR (<10 min) [43,50,67]. Absolute quantitation of lipids using NMR remains a challenge [68].

High resolution NMR has been shown to detect a variety of diseases directly in human tissue and fluid samples [69]. Portable NMR-based biosensors have also been developed for use as medical diagnostics [70]. However, portable NMR sensors suffer from low sensitivity and specificity while high-resolution NMR tools require expensive equipment and trained personnel to analyze results.

### 2.4. Biosensors

**Figure 1:**
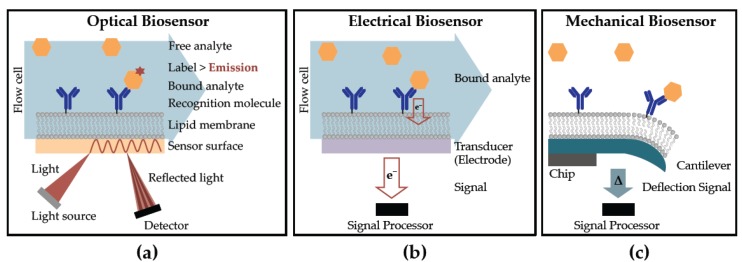
Examples of biosensor techniques incorporating lipids for the detection of analytes include (**a**) optical (**b**) electrical and (**c**) mechanical.

Broadly defined, biosensors recognize target molecules and produce a measurable signal. Optical, electrochemical and mechanical biosensors (Figure 1) have been adapted for the detection of lipidic and amphiphilic biomarkers, using a variety of assay methodologies which can be categorized as (a) labeled and (b) label-free. Labeled assays indirectly measure binding of an analyte to the target molecule, using a reporter molecule (an indicator). Labeled assays have the advantage of readily multiplexing, and are highly desirable in clinical applications. Label-free assays measure signal changes directly associated with either target binding or cellular processes, without the need for an external reporter. The advantages of label-free biosensors include reduced assay complexity, cost and decreasing interference of the label on binding between ligand and target.

#### 2.4.1. Optical Biosensors Detecting Lipids

Optical biosensors measure binding-induced changes in light from the sensor surface. Surface plasmon resonance (SPR) is a label-free technology that has been used to detect the interaction of amphiphilic molecules with lipid bilayers, for ligand screening and biomarker discovery [71]. In SPR sensing, a ligand is immobilized on a gold-coated surface while an interacting molecule is injected in aqueous solution, and the change in resonance associated with binding is measured optically using a spectrometer. SPR has been optimized for measuring protein-protein affinities using the BIAcore™ (GE Healthcare) system. In original iteration, SPR systems lack resolution, cannot multiplex, and are difficult to miniaturize. However, several researchers are working on overcoming these limitations, which may provide for more robust sensors in the future [72]. SPR is not conducive for detection of amphiphiles in biologically relevant conditions, as required for diagnostic applications.

Interferometry, particularly Backscattering interferometry (BSI), allows for label-free detection of both surface-bound and free-solution molecules [73]. In BSI, excitation of a microfluidic channel containing the sample, as well as the channel surface, usually made of polydimethylsulfoxide or glass, yields interference fringes. Changes in fringes associated with binding of two molecules are measured in the backscatter region. BSI detects interactions of amphiphiles with lipid bilayers [74] directly in human serum [75]. However, in complex samples, non-specific interactions and associated changes in refractive index can prove challenging, but can be limited using suitable surface functionalization chemistry [76]. BSI is inexpensive, sensitive and very versatile, making it a promising candidate for detection of amphiphilic biomarkers in patient samples.

Ellipsometry measures the refractive index of a thin film, and has been utilized in label-free assays where changes in refracted light are measured upon interaction of giant lipid vesicles with a poly-l-lysine coated surface, using a biosensor based on total internal reflection imaging ellipsometry (TIRIE) [77]. This sensor can detect µm size particles such as cells, capsules and liposomes. However, the ability to detect such particles in tissues, as well as the overall clinical utility of this system is yet to be demonstrated.

An optical assay based on UV absorption of lipid-functionalized gold nanorods has been used to detect the lipopeptide myristoyl-Lys-Arg-Thr-Leu-Arg, and a variety of other lipid biomarkers, in serum [78]. However, the low specificity of this label-free technique requires mass spectrometry analysis in order for multiplexing to take place.

**Figure 2:**
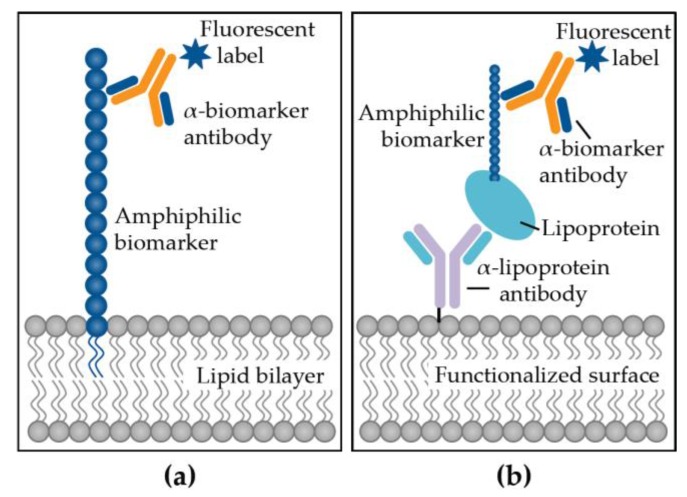
Immunoassay strategies to detect lipid and amphiphilic biomarkers using (**a**) membrane insertion or (**b**) lipoprotein capture.

In labeled assays, the target molecule is immobilized on the surface of a biosensor and then probed with an analyte, typically an antibody, coupled to a label (fluorophore, quantum dot, radioisotope, enzyme) [79]. Mukundan et al. have employed an optical waveguide-based biosensor measuring only surface-bound fluorescent signals from labeled antibodies for the sensitive and specific detection of lipid and amphiphilic targets directly in clinical samples [80,81,82]. This system utilizes the interactions of these biomarkers with lipid bilayers and lipoproteins to capture them directly to the waveguide surface, followed by probing with labeled-antibodies excited by waveguide-coupled laser light (Figure 2). This system has been demonstrated to effectively detect amphiphilic biomarkers associated with *Mycobacterium tuberculosis* [15,83], *M. bovis* [84], *Escherichia coli* [85], *Salmonella* Typhimurium [86], influenza [87], tumor markers [88,89] oftentimes directly in human serum, proving potential utility as a diagnostic tool [80,82]. These assays have been multiplexed for the simultaneous detection of several biomarkers [81]. However, the main disadvantage of using labeled reagents is the time and cost of the labeling process, and shelf life of the labeled ingredients [90].

#### 2.4.2. Electrochemical Biosensors Detecting Lipids

Electrochemical sensing is a label-free method where an electrode is used to directly detect associated reactions [91]. Electrochemical biosensors have had the greatest commercial success (glucose monitors) due to their low-cost, ease of use and miniaturization properties [92]. Amperometric sensors measure current typically as a result of electron transfer during the binding between a molecule, and chemically functionalized surface, as exemplified in the detection of LPS using redox diacetylenic vesicles on a sol-gel thin-film electrode [93]. Another example is the binding of cholera toxin to a glycosphingolipid ganglioside GM1 coated gold surface, which has been developed into a sensitive, low-cost sensor [94]. Potentiometric sensors measure potential or charge accumulation and have been used to detect lipid antigens [95] such as amphiphilic cholesterol using lipid films [96,97], without interference from ascorbic acid, glucose, urea or other proteins and lipids. In addition, label-free electrochemical biosensors have been developed for the detection of lipids such as low-density lipoprotein (LDL) with high sensitivity and specificity [98,99,100]. These sensors have also displayed long-term stability and high reproducibility with small sample volumes, which make for a promising clinical tool. However, a problem with electrochemical detection of biomarkers is specificity of detection in complex biological samples, as these platforms are more sensitive to even small perturbations in the background, which is always a possibility in clinical measurements.

#### 2.4.3. Mechanical Biosensors Detecting Lipids

Mechanical biosensors provide rapid and sensitive measurements without extensive sample processing, ideal for clinical application. The two main mechanical sensing techniques are based on cantilever and quartz crystal microbalances (QCM), both of which are label-free. QCM detects changes in resonance frequency on the sensor surface from increased mass due to analyte minding [92]. QCM has been used to observe the formation of supported lipid bilayers in real-time [101] and lipid exchange between a bilayer and vesicle [102]. The latter measures mechanical bending of a receptor-functionalized microcantilever upon binding of the target molecule, as demonstrated for LPS [103]. Another example is the measurement of the interaction between amyloid-β and the cell membrane as an early indicator of Alzheimer's disease [104]. A major drawback to cantilever sensors is that they often operate in air, rather than liquid samples, limiting their clinical utility.

## 3. Lipid Biomarkers for Infectious Diseases

Rapid diagnosis is important to halt the spread of both endemic and emerging pathogens, as well as multi-drug resistant organisms [105]. Infectious diseases are caused by bacterial, viral, fungal and parasitic organisms and are routinely diagnosed using culture, microscopy, serology and genetic tests [106], all of which are time-consuming and performed by trained laboratory personnel. Several biosensors have been developed for diagnosis of infectious diseases [11], however all come with challenges that limit their application in the clinic. Many biomarkers associated with infectious diseases are amphiphiles, which are difficult to detect, especially in aqueous blood (Table 2).

### 3.1. Sepsis

Sepsis is an infection of the bloodstream resulting in uncontrolled activation of the immune system [115]. Morbidity and mortality results from organ failure, which can be avoided with early and effective treatment, which in turn, requires rapid diagnostics [116]. There are several lipid and amphiphilic biomarkers associated with sepsis. LPS (also known as endotoxin) is a classic pathogen associated molecular pattern (PAMP) shed by Gram-negative bacteria. LPS activates the innate immune receptor, Toll-like Receptor 2, resulting in a cytokine cascade, which is the mechanism of endotoxic shock [117]. The presence of LPS in patient blood is a clear indicator of sepsis. However, detection of LPS in aqueous blood is complicated by the molecule’s amphiphilic biochemistry, which drives it to associate with host carrier lipoproteins [118] and other molecules such as LPS-binding protein (LBP), high-density lipoprotein (HDL), low-density lipoprotein (LDL), very low-density lipoprotein (VLDL) and bactericidal/permeability-increasing protein [109]. Similarly, LTA is an amphiphilic cell wall component of Gram-positive bacteria whose presence in blood also indicates sepsis. *Staphylococcus aureus* LTA in human blood preferentially binds to HDL (68%), then to LDL (28%), and minimally to very low density lipoprotein (4%) [112]. LBP also binds to LTA [109]. These associations complicate the detection of amphiphilic LPS and LTA in blood.

### 3.2. Mycobacterial Infections

Lipoarabinomannan (LAM) is a biomarker for diagnosis of tuberculosis (TB) [119,120,121,122]. LAM is an amphiphilic heat stable lipoglycan, which forms an integral component of the mycobacterial cell wall [119,120], and is secreted during TB infection. LAM has proved to be an excellent diagnostic target especially in smear negative patients (HIV-positive, pediatric population and others). Some limitations of LAM include assay cross reactivity with common oral flora like Candida and Actinomyces that can result in lower predictive value of detection [123]. LAM assays can be improved using standardized sample processing methods and more sensitive sensor technology, as well as more specific antibodies [124]. LAM is readily detected in urine [88,89], but not in blood, likely because it associates with HDL in blood [15]. To this end, lipoprotein capture assays that utilize antibodies to target HDL have been used to pull down bound LAM in serum samples [15,83,84]. This method targets host pathogen interactions, and can be applied to other amphiphilic biomarkers in blood [83]. Pathogen biomarker based measurements can be used in both human and animal hosts [84,113]. However, the high cost of such technology, and biochemical nature of LAM are current challenges to the development of such assays [124].

### 3.3. Antimicrobial Resistance

The global spread of antimicrobial resistance underlines the need for rapid and accurate assays for the determination of resistant organisms prior to antibiotic treatment. A common mechanism of antibiotics is inhibition of bacterial cell wall components, which are lipid or amphiphilic molecules [125]. Modifications of membrane lipids are common mechanisms of antimicrobial resistance in bacteria [126,127,128] and the direct detection of such changes would support effective treatment. In fact, targeting PAMPs that are highly conserved, such as membrane lipids, for molecular diagnostics has a tremendous benefit over approaches targeting more rapidly evolving aspects of bacterial physiology (i.e., PCR). MALDI-TOF MS has been used to detect antibiotic resistance caused by lipid alterations [111]. E.g., detection of LPS alterations that reduce the molecules net negative charge resulting in resistance to antimicrobial peptides such as colistin, and OmpK36 porin loss in *Klebsiella pneumoniae* resulting in resistance to carbapenems. However, the initial cost of setting up a clinical MALDI-TOF system is prohibitive toward application in routine diagnostic settings.

### 3.4. Malaria

Hemozoin is an insoluble amphiphilic crystalline byproduct formed from the degradation of blood by parasites such as *Plasmodium falciparum*, the causative agent of malaria [114]. Hemozoin is released into circulation upon erythrocyte lysis, and its presence in patient blood is a direct indication of malarial infection [129]. Detection of hemozoin has mostly been achieved using microscopy and flow cytometry in blood [130]. The amphiphilic nature of hemozoin hampers its direct detection in blood due to sequestration by host immune components [129] such as apolipoprotein E and LBP [114]. Methods like Lipoprotein capture can potentially be adapted for the detection of amphiphilic hemozoin in blood.

## 4. Lipid Biomarkers for Non-infectious Diseases

Non-infectious diseases including cancer and cardiovascular disorders are a result of a malfunction in normal physiological processes. Therefore, identifying specific biomarkers for diagnosing non-infectious diseases presents a major challenge. Rather than targeting a foreign molecule that is absent in a healthy patient, the diagnosis of non-infectious diseases often measures irregularities in expression of host-derived molecules [131,132]. Many lipid and amphiphilic molecules associated with human metabolism can be used as biomarkers when differentially detected in diseased versus healthy individuals (Table 3). Detection of such biomarkers can potentially be used to determine prognosis, monitor recurrence, evaluate disease progression, and predict a patient’s risk of developing a particular disease [133].

### 4.1. Cardiovascular Diseases and Disorders

Cardiovascular diseases (CVD) [155] account for 17.5 million deaths, accounting for 31% of all deaths worldwide [155]. Heart attacks and strokes, mainly caused by a blockage that prevents blood from flowing to the heart or brain account for about 80% of all CVD deaths. In many cases, dyslipidemia, or abnormal levels of lipids in the blood, is a major mediator of CVD [156,157]. It is typically characterized by increased levels of total cholesterol, increased levels of low density lipoprotein cholesterol (LDL-C), increased levels of TG, decreased levels of high density lipoprotein cholesterol (HDL-C), modified function of lipid molecules, or a combination of some or all of these factors [156,157,158]. Cholesterol is a major component of cell membranes, and contributes to their structural integrity and fluidity [131]. Cholesterol is also a precursor of vitamin D and important steroid hormones such as progesterones, glucocorticoids (cortisol), androgens (testosterone) and estrogens [131,159]. Cholesterol homeostasis is critical to cardiovascular health. High cholesterol can raise CVD risk [135,160], by creation of sticky plaque deposits along arterial walls, eventually blocking blood flow [125,160], causing heart attacks and strokes. LDL and HDL are important carriers of cholesterol. Increased levels of LDL-C can cause cholesterol buildup in arteries, resulting in their narrowing, raising cardiovascular risk [161]. HDL is a scavenger, carrying cholesterol away from the arteries and back to the liver for breakdown [134]. A high TG level combined with high LDL-C and low HDL-C is linked with fatty buildups in artery walls [132,149,150].

Assessing CVD risk largely involves serum lipid profiling, focused on measuring total cholesterol, HDL-C, LDL-C, and TG levels [162], largely by use of labeled optical immunoassay platforms. For instance, the Alere Cholestech LDX^®^ System combines enzymatic optical detection with solid-phase technology to rapidly and sensitively measure total cholesterol, HDL cholesterol, triglycerides and glucose in a finger prick of blood [163]. Cholesterol is measured enzymatically in serum in a series of coupled reactions that hydrolyze cholesteryl esters and oxidize the 3-OH group of cholesterol, byproducts of which are measured colorimetrically [164,165,166]. TGs are measured enzymatically in serum via a series of coupled reactions that produce glycerol, which is oxidized using glycerol oxidase. H_2_O_2_, one of the reaction products, is measured colorimetrically [164]. In clinical practice, the standard measure of HDL is quantification of its cholesterol content after precipitation of apolipoprotein B (coat protein for LDL) [164]. Other techniques to determine HDL-C in serum include ultracentrifugation, electrophoresis, HPLC, precipitation-based method, direct measuring methods, and NMR [164,167].

LDL-C is often determined using the “Friedewald formula” which incorporates measured values for total cholesterol, HDL-C, and TG as: LDL-C = (total cholesterol) – (HDL-C) – (TG/5) [168]. This equation assumes that the ratio of TG to cholesterol is constant, which is not always the case. The Friedewald formula can thus underestimate LDL-C at lower levels of LDL-C, and higher levels of TG (>400 mg/dL) [169]. Modifications to the Friedewald formula have tried to address these shortcomings [169]. Direct LDL-C measurements (e.g., ultracentrifugation, electrophoresis, chemical precipitation, immunoseparation, and homogenous assays) are available, but standardization and extensive validations are needed for use in clinical measurements [169]. For clinical sampling, serum lipid serum profiles are determined using diagnostic test kits, and analyzed with chemical automated continuous flow analyzers that use photometric and colorimetric testing (Hitachi 7180/7020 Hitachi Clinical Analyzers, Randox RX Series Clinical Chemistry Analyzers), and point-of-care devices (Alere Cholestech LDX microfluidic device system, Professional CardioChek PA test strip system, Accutrend Plus test strip system) [170].

Many CVDs cannot be explained by these current standards [171], as many patients have lipid levels within the recommended range. Thus, there is a need for additional biomarkers for early diagnosis and prevention of CVD. Several biomarkers have been identified and are being explored for diagnostic potential. Cardiolipin is one such biomarker, reduced concentrations and altered composition of which have been implicated in cardiomyopathy associated with Barth Syndrome [172,173]. Diabetic cardiomyopathy is characterized by altered lipid composition and mitochondrial dysfunction [173,174] and reduced cardiolipin metabolism has been implicated here as well. Mitochondrial dysfunction due to reactive oxygen species effect has been shown to be associated with cardiac ischemia/reperfusion [21,23]. Mitochondrial cardiolipin have been proposed to undergo lipid peroxidation because of either their high content of unsaturated fatty acids or because of their location in the mitochondria [173,175]. Oxidized cardiolipin, a natural antigen that has pro-inflammatory effects, is also associated with atherosclerosis [173,176]. Current methods to detect the pro-inflammatory effects and thrombosis induced by cardiolipin rely on determination of antibodies against oxidized cardiolipin with commercially available standard enzyme-linked immunosorbent kits (e.g., Orgentec Anti-Cardiolipin Screen, Mainz, Germany) [173,176].

### 4.2. Cancer

Cancer is the leading cause of death worldwide, with 8.8 million deaths in 2015 [155]. Most cancers are initially diagnosed either because of the appearance of signs or symptoms of the disease, or via screening, which is not always available [177]. Definitive diagnosis is by biopsy of the affected tissue. The main treatments for many cancers are surgery, chemotherapy, and radiation therapy. The effectiveness of these treatments depends on early diagnosis, which results in a higher chance of survivability [177]. Therefore, there is a need for more research on molecular diagnostics based screening and early detection of cancer.

Dyslipidemia has been proposed to have an association with an increased risk of cancer, particularly breast and prostate cancers. Some studies exploring causal associations between serum lipids and breast cancer have observed increased levels of total cholesterol, TG, and LDL-C and decreased levels of HDL-C [136,137,139,142]. However, these trends are not always consistent [178,179]. Similar observations were made for prostate cancer, with some studies showing an abnormal lipid trend, similar to breast cancer [136,138,140,141,143]. Other studies found no correlation or opposite trends [180,181,182]. Thus, we clearly do not have a good understanding of the pathophysiological effects of how lipids levels contribute to cancer. Methods to detect serum lipid levels are similar to those that are currently being applied to assessing cardiovascular disease, described in the CVD section [170].

Nobel laureate Otto Warburg claimed that cancer originated from irreversible injury to mitochondrial respiration, the structural basis for which is still unclear [183,184]. A possible hypothesis is that mitochondrial dysfunction induced by oxidative stress can cause lipid peroxidation that contributes to the development of cancer [37]. One of the culprits implicated as a candidate for lipid peroxidation is cardiolipin [37]. Kiebish et al. found abnormalities in cardiolipin content or composition in several types of mouse brain tumors, compared to normal brain [37]. These abnormalities were closely associated with significant reductions in energy-generating activities [37]. ESI-MS showed that the cardiolipin composition was significantly different in prostate tissue isolated from normal individuals versus cancer patients [154].

More discovery efforts need to be undertaken to understand the roles lipids play in cancer [185,186,187,188]. Most of the findings suggest that there are abnormal levels of lipids- phosphatidylcholines, phosphatidylethanolamines, lysophosphatidylcholine, lysophosphatidic acid, LysoPI, ceramides, cholesteryl oleate- in biological samples from cancer patients than in normal ones [185,186,187,188].

Detection of lipidated biomarkers has been explored for many cancers. For instance, the carcinoembryonic antigen (CEA) has been implicated in a variety of cancers such as breast, colorectal, pancreatic and others [189,190,191]. Detection of CEA is mainly performed using targeted antibodies, and optical detection platform—labeled immunoassays. Studies have found that serial CEA measurements can detect recurrent colorectal cancer with ~80% sensitivity and 70% specificity, 5 months in advance of other diagnostics! CEA has been suggested to be the most frequent indicator of recurrence in asymptomatic patients and detection of this biomarker is the most cost-effective test for the preclinical detection of resectable disease. CEA is most useful for the early detection of liver metastasis in patients with diagnosed colorectal cancer. The biomarker is also valuable as a prognostic indicator for breast cancer [192]. In addition to optical colorimetric measurements, radioimmunoassays have also been used for CEA measurement [193,194,195,196,197,198]. Our team has developed and validated the measurement of CEA in serum and nipple aspirated fluid from patients with abnormal mammograms using a fluorescence sandwich immunoassay on a waveguide-based optical biosensor platform, demonstrating picomolar sensitivity of detection of the antigen [88,89]. The challenges with CEA detection include the fact that the biomarker has been implicated with other non-cancerous conditions such as smoking, and that the presence of the biomarker in blood is not specific indicator of a type of cancer.

Another significant cancer biomarker family, the Carbohydrate antigens, are also amphiphilic and belong to the mucin-class of molecules, which promote cancer cell proliferation and inhibits anti-cancer immune responses [199]. Of these, CA125 is best known as a biomarker to monitor epithelial ovarian cancer and for the differential diagnosis of pelvic masses [200,201]. Serum levels of CA125 are routinely monitored in patients with ovarian cancer as prognostic indicators of cancer recurrence. Genway Biosystems has developed a colorimetric immunoassay for this biomarker, which is just one of several such assays available for this target [202]. CA15-3 (MUC1) is yet another biomarker, which has been extensively implicated in a variety of cancers, and is routinely used as a prognostic indicator, and a measure of cancer severity [203]. Again, immunoassay methods, including radioimmunoassay methods are used for the measurement of these antigens in serum. These assays lack sensitivity and specificity, and are associated with a high false-positive rate, which needs to be addressed to increase their clinical utility. For example, Roche Laboratories has developed an electro-chemiluminescence assay for the detection of CA15-3 in blood [204]. These immunoassays utilize a ruthenium-complex and tripropylamine. The chemiluminescence reaction for the detection of the reaction complex is initiated by applying a voltage to the sample solution resulting in a precisely controlled reaction.

Detection of exosomes has been explored for many cancers. Exosomes are small heterogeneous extracellular vesicles (40–150 nm) released by most cell types in bodily fluids such as urine, plasma, saliva and breast milk [205,206]. While their exact function is still relatively unknown, the current theories are that they are involved in intracellular communication and cellular waste disposal [205,206]. The lipid composition of exosomes includes many different lipid classes as well as DNAs, RNAs and proteins. The exosomes are believed to contain the molecular constituents of the cell that they were derived from. In particular, exosomes have been identified as a potential biomarker for early detection and prognosis of cancer as they promote tumorigenesis, growth, progression and metastasis [205,206,207,208]. Exosome secretion by cancer cells is considerably up regulated compared to non-cancerous cells [1,2,3,4]. RNAs (messenger RNA, microRNA, long non-coding RNA), DNAs (mitochondrial DNA, single stranded DNA, double stranded DNA), and proteins (small Rab family GTPases, annexins, survivin, CD9, CD 24 and CD34) are endosomal payloads that have been found to be elevated or altered in cancer cells compared to non-cancerous cells [207,208,209]. Quantitative real-time polymerase chain reaction, nucleic acid sequencing, antibody-based methods for detection and quantitation (e.g., Western blot, ELISA), nanoparticle tracking, dynamic light scattering, flow cytometry, transmission electron microscopy have all been applied to the detection and quantification of endosomes as well as the characterization of the contents of the endosomes [207,208,209].

### 4.3. Preeclampsia

Preeclampsia (PE) is a complex pregnancy disorder that is characterized by hypertension and proteinuria [210]. PE affects 2%–8% of pregnancies worldwide [211]. It is the most common pregnancy complication and is associated with high maternal and perinatal mortality. PE may be life-threatening for both mother and child, increasing both fetal and maternal morbidity and mortality [212,213]. In the mother, PE may cause premature cardiovascular disease, such as chronic hypertension, ischemic heart disease, and stroke [212,213]. Children birthed from preeclamptic pregnancies have an increased risk of stroke, coronary heart disease, and metabolic syndrome [212,213]. While the pathophysiology of PE remains unclear, this disorder is mediated abnormal placentation that trigger endothelial dysfunction, resulting in vasoconstriction, thrombosis, and end-organ ischemia [210]. Dyslipidemia has been shown to be associated with an increased risk of preeclampsia [147,214]. Several meta-analysis studies, which involved PE case-control studies as well as prospective cohort studies show that while this abnormal lipid pattern may involve increased TC, TG, LDL-C and decreased HDL-C, the results are not always consistent [144,145,146,147]. However, in many of these studies there seems to be a clear consensus of raised TC levels, resulting in hypertriglyceridemia [144,145,146,147]. It is still unclear whether hypertriglyceridemia is a risk factor for preeclampsia or whether there is any causal association between them [145,146,147]. More studies need to be done to understand the role maternal TG plays the pathophysiology of PE. Because PE display an abnormal serum lipid profile, many of the bioassays and methods developed for CVD are readily applied to test for PE. These bioassays and methods have been discussed in the CVD section. Recent mass spectrometry based lipidomic studies have revealed several potential lipid biomarkers associated with PE [34,215]. These include oxidized cholesterol, cholesteryl ester, oxidized sphingomyelin, ceramide, glycerophosphocholine, and lysophosphatidylcholines.

### 4.4. Lipotoxicity

Lipotoxicity is a metabolic disorder characterized by excessive accumulation of fatty acids within the cell and has been implicated in the development of heart failure, obesity and diabetes [216,217]. Adipose tissues play a critical role in energy storage [218]. Adipocytes, the primary cells forming adipose tissue, contain cytosolic lipid droplets composed of a core of neutral lipids, including sterol esters and TGs, surrounded by a phospholipid monolayer [218,219,220,221,222,223,224]. Lipotoxicity is the abnormal accumulation of lipid droplets in non-adipose tissues, which leads to mitochondrial dysfunction, inhibition of ATP generation, and ultimately cell death by apoptosis [217,225,226]. Manifestation of lipotoxicity typically occurs in kidney, liver, heart and skeletal muscle [217]. For example, a disease estimated to affect 10 to 24% of the global population [227,228] is the accumulation of fat within the liver termed nonalcoholic fatty liver disease (NAFLD) [229]. NAFLD is characterized by increased hepatocyte accumulation of TGs within the cytosol [230] and can progress from fatty liver accumulation to liver fibrosis and cirrhosis [227,231]. Serum TG and cholesterol have been implicated as potential biomarkers for lipotoxicity and are currently detected using serum lipid profiling as described in the CVD section [148,151,152,170].

## 5. Discussion and Conclusions

**Figure 3:**
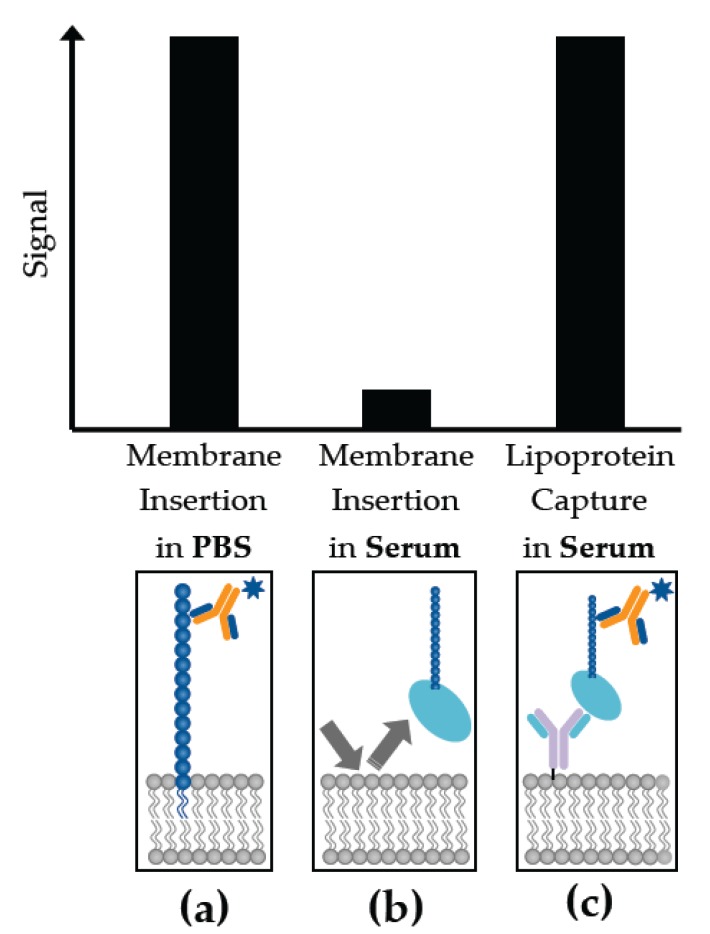
A comparison of the sensitivity of membrane insertion and lipoprotein capture technologies for the measurement of amphiphilic biomarkers based on the medium of presentation (serum vs. buffer). Biomarkers that are readily detected in (**a**) phosphate buffered saline (PBS) can be poorly observed in (**b**) serum when utilizing a membrane insertion assay strategy, because of the uptake of these signatures by host serum carrier molecules. This signal can be recovered if (**c**) lipoproteins are incorporated in the assay format, exploiting the host pathogen interaction for maximal sensitivity of detection.

Amphiphilic and lipidic molecules are significant mediators in a variety of biological processes associated with infectious diseases and non-infectious conditions such as cancer and CVD. Many of the PAMPs secreted by bacteria are amphiphilic, as are a significant proportion of biomarkers associated with neurological processes. Many cancer signatures are also lipidated. Thus, ignoring this unique category of molecules will only limit our understanding of host-pathogen biology and disease processes. Because of the challenges in raising antibodies to such targets, and developing sensitive labeled assays, many of these biomarkers are not efficiently used in the diagnosis of disease. The biochemistry of these molecules should be considered in the design and execution of these assays. For instance, the conformation of the amphiphilic biomarkers changes dramatically depending on the milieu in which they are present (Figure 3), and the critical micelle concentrations of these molecules [232]. Lipidated biomarkers adhere to plastic surfaces, decreasing the sensitivity of their detection [233]. These characteristics also manifest themselves in physiological samples such as blood. Amphiphilic molecules, depending on their critical micelle concentration, will either self-aggregate into micelles, or associate with carrier moieties such as HDL and LDL, making their direct and rapid detection more challenging (Figure 3). Understanding the biochemical characteristics of the amphiphilic targets and anticipating the impact of these features in physiological systems can allow for the development of tailored, sensitive assays for their detection.

## Figures and Tables

**Table 1:** Advantages and limitations of sensors as clinical diagnostic tools for detecting lipid and amphiphilic biomarkers.

Method	Advantages	Limitations
**Mass spectrometry**	Sensitive	Expensive
	Specific	Sample preparation can be extensive
	Works in patient samples	Requires highly trained personnel
		Requires laboratory infrastructure
**NMR-based sensors**	Rapid	Low sensitivity
	Reproducible	Low specificity
	Works in patient samples	Sample preparation can be extensive
		Requires highly trained personnel
		Requires laboratory infrastructure
**Optical biosensors**		
SPR-based sensors	Rapid	Low sensitivity in patient samples
	Specific	
Interferometry-based sensors	Low-cost	Low specificity in patient samples
	Sensitive	
Waveguide-based sensors	Rapid	Short shelf-life of labeled reagents
	Reproducible	
	Sensitive	
	Specific	
	Works in patient samples	
**Electrochemical biosensors**	Rapid	Low sensitivity in patient samples
	Reproducible	Low specificity in patient samples
**Mechanical biosensors**	Rapid	Low sensitivity in patient samples
		Low specificity in patient samples
		Reproducibility

**Table 2:** Select lipid and amphiphilic biomarkers used for the diagnosis of infectious diseases.

Biomarker	Disease	Location	Interacting Molecules	Reference
Lipopolysaccharide (LPS)	Sepsis	Blood	LBP, HDL, LDL	[107,108]
			holotransferrin	[109]
	Urinary tract infection	Urine	n.d.	[110]
	Antimicrobial resistance	Any sample	n.d.	[111]
Lipoteichoic acid (LTA)	Sepsis	Blood	HDL, LDL, VLDL	[108,112]
			LBP, holotransferrin	[109]
Lipoarabinomannan (LAM)	Tuberculosis	Urine	n.d.	[113]
		Blood	HDL	[15]
Lipomannan (LM)	Bovine tuberculosis	Blood	HDL	[84]
OmpK36 porin	Antimicrobial resistance	Any sample	n.d.	[111]
Hemozoin (HZ)	Malaria	Blood	LBP, HDL, LDL, VLDL, apolipoprotein E, α-1-antitrypin	[114]

LBP: LPS-binding protein; HDL: high-density lipoprotein; LDL: low-density lipoprotein; VLDL: very low density lipoprotein; n.d.: not determined.

**Table 3:** Select lipid and amphiphilic biomarkers used for diagnosing non-infectious diseases.

Biomarker	Disease	Location	Interacting Lipoproteins	Reference
Cholesterol	Cardiovascular disease	Blood	HDL, LDL	[134,135]
	Cancer	Blood	HDL, LDL	[136,137,138,139,140,141,142,143]
	Preeclampsia	Blood	HDL, LDL	[144,145,146,147]
	Lipotoxicity	Blood	HDL, LDL	[148]
Triglycerides (TG)	Cardiovascular disease	Blood	LDL, VLDL	[132,149,150]
	Cancer	Blood	LDL, VLDL	[136,137,138,139,140,141,142,143]
	Preeclampsia	Blood	LDL, VLDL	[144,145,146,147]
	Lipotoxicity	Blood	LDL, VLDL	[148,151,152]
Cardiolipin (CL)	Cardiovascular disease	Blood	LDL, HDL, VLDL	[153]
	Cancer	Brain tissue, Prostate tissue	n.d.	[37,154]

HDL: high-density lipoprotein; LDL: low-density lipoprotein; VLDL: very low density lipoprotein; n.d.: not determined.
